# Bone marrow uptake of ^18^F-fluorodeoxyglucose in Hodgkin lymphoma without bone involvement: comparison between patients with and without B symptoms

**DOI:** 10.1590/0100-3984.2016.0201

**Published:** 2018

**Authors:** Rômulo Hermeto Bueno do Vale, Daniela Andrade Ferraro, Paulo Schiavom Duarte, Giovana Carvalho, Marcos Santos Lima, George Barbério Coura Filho, Marcelo Tatit Sapienza, Carlos Alberto Buchpiguel

**Affiliations:** 1 MD, Division of Nuclear Medicine, Instituto do Câncer do Estado de São Paulo (Icesp), São Paulo, SP, Brazil.; 2 MD, PhD, Division of Nuclear Medicine, Instituto do Câncer do Estado de São Paulo (Icesp), São Paulo, SP, Brazil.

**Keywords:** Fluorodeoxyglucose F18, Positron emission tomography computed tomography/methods, Hodgkin disease, Bone marrow/diagnostic imaging, Fluordesoxiglucose F18, Tomografia computadorizada com tomografia por emissão de pósitrons/métodos, Doença de Hodgkin, Medula óssea/diagnóstico por imagem

## Abstract

**Objective:**

To compare the degree of benign bone marrow uptake of ^18^F-fluorodeoxyglucose (^18^F-FDG) between Hodgkin lymphoma patients with and without B symptoms.

**Materials and Methods:**

We analyzed the medical charts of 74 Hodgkin lymphoma patients who underwent ^18^F-FDG positron emission tomography/computed tomography (PET/CT) prior to the initiation of therapy between October 2010 and September 2013. In all of the patients, the bone marrow biopsy was negative and the ^18^F-FDG PET/CT images did not suggest bone marrow involvement. Of the 74 patients evaluated, 54 presented inflammatory (B) symptoms and 20 did not. Regions of interest (ROIs) were drawn on the sternum, the proximal thirds of the humeri, the proximal thirds of the femora, and both iliac wings (totaling seven ROIs per patient). To compare the patients with and without B symptoms, in terms of standardized uptake values (SUVs) for the seven ROIs, we used the Mann-Whitney U test.

**Results:**

For six of the ROIs, the SUVs were higher in the patients with B symptoms than in those without, and the difference was statistically significant (*p* < 0.05). There was also a tendency toward a statistically significant difference between the two groups in terms of the SUV for the right iliac wing ROI (*p* = 0.06).

**Conclusion:**

In our sample, the presence of B symptoms was associated with increased ^18^F-FDG uptake in bone marrow.

## Abstract (pt)

**Objetivo:**

Comparar o grau de absorção benigna de ^18^F-fluordesoxiglicose (^18^F-FDG) na medula óssea de pacientes com linfoma de Hodgkin com e sem sintomas B.

**Materiais e Métodos:**

Analisamos os prontuários de 74 pacientes com linfoma de Hodgkin submetidos a tomografia por emissão de pósitrons/tomografia computadorizada (PET/CT) com ^18^F-FDG antes do início da terapia entre outubro de 2010 e setembro de 2013. Em todos os pacientes, a biópsia da medula óssea foi negativa e as imagens de ^18^F-FDG PET/CT não sugeriram envolvimento da medula óssea. Dos 74 pacientes avaliados, 54 apresentaram sintomas inflamatórios (B) e 20 não. As regiões de interesse (ROIs) foram desenhadas no esterno, nos terços proximais dos úmeros, nos terços proximais dos fêmures e nas duas asas ilíacas (totalizando sete ROIs por paciente). Para comparar os pacientes com e sem sintomas B, em termos dos *standardized uptake values* (SUVs) para as sete ROIs, utilizamos o teste U de Mann-Whitney.

**Resultados:**

Para seis das ROIs, os SUVs foram maiores nos pacientes com sintomas B do que nos pacientes sem, e a diferença foi estatisticamente significante (*p* < 0,05). Houve também tendência para uma diferença estatisticamente significante entre os dois grupos em termos do SUV para a ROI da asa ilíaca direita (*p* = 0,06).

**Conclusão:**

Na nossa amostra, a presença de sintomas B foi associada ao aumento da captação de ^18^F-FDG na medula óssea.

## INTRODUCTION

Hodgkin lymphoma accounts for approximately 12% of all cases of lymphoma and 1% of all malignancies^(1)^. The therapies used in the initial treatment depend on the stage of the disease at diagnosis^(2-4)^. Therefore, appropriate staging before the initiation of therapy is crucial^(5)^. Lymphoma staging, which is based on the Ann Arbor system^(6,7)^, usually involves computed tomography and bone marrow biopsy.

Functional imaging employing ^18^F-fluorodeoxyglucose positron emission tomography/computed tomography (^18^F-FDG PET/CT) has come into widespread use in the management of Hodgkin lymphoma. Because ^18^F-FDG PET/CT is accurate at differentiating residual viable tumor from therapy-induced fibrosis^(8)^, it has been incorporated into the recently revised criteria for end-of-therapy assessment^(9)^. In addition, various studies have suggested that ^18^F-FDG PET/CT can assess the treatment response early in the course of therapy for Hodgkin lymphoma, thus allowing the therapy to be tailored to each patient, on the basis of the individual risk of relapse^(10,11)^. Data from a baseline examination increases the accuracy of the ^18^F-FDG PET/CT assessment of the treatment response Performing ^18^F-FDG PET/CT at baseline has therefore been strongly encouraged in cases of Hodgkin lymphoma. A pre-treatment ^18^F-FDG PET/CT scan can also provide information that is useful for the initial staging^(12)^ and for the implementation of radiotherapy^(13)^.

Diffuse uptake of ^18^F-FDG in the axial skeleton has been described in cases of diffuse bone marrow infiltration of malignant lymphoma^(14)^ and diffuse bone marrow metastases^(15)^. Benign diffuse bone marrow ^18^F-FDG uptake secondary to bone marrow stimulation by granulocyte-macrophage stimulating factor^(16)^, granulocyte colony- stimulating factor, or erythropoietin^(17)^ has also been reported. There have also been reports of diffuse bone marrow ^18^F-FDG uptake resulting from hematologic diseases, including chronic myeloid leukemia^(18)^ and myelofibrosis^(19)^. However, we have noted diffuse bone marrow uptake in some patients before the onset of treatment, without bone marrow infiltration and without the use of granulocyte colony-stimulating factor or erythropoietin. We hypothesized that this uptake would be associated with the presence of inflammatory (B) symptoms. The objective of this study was to compare the degree of diffuse benign bone marrow uptake of ^18^F-FDG in Hodgkin lymphoma patients with and without B symptoms.

## MATERIALS AND METHODS

### Patient population

We reviewed the medical charts of 74 Hodgkin lymphoma patients who underwent ^18^F-FDG PET/CT studies prior to the initiation of therapy, between October 2010 and September 2013. All patients had a negative bone marrow biopsy and ^18^F-FDG PET/CT images that were not suggestive of bone marrow involvement. Therefore, given that both methods are complementary for the diagnosis of bone marrow involvement^(20)^, the patients were considered free of bone marrow disease. The following ^18^F-FDG PET/CT patterns were considered suggestive of bone marrow infiltration: focal, multifocal, or heterogeneous bone marrow uptake; and any suspicious alterations on the CT scan. Patients presenting any pattern suggestive of bone marrow infiltration were excluded from the analysis. One patient with a femoral prosthesis was also excluded because the prosthesis produced an artifact in the images, impairing the local analysis. Of the 74 patients evaluated, 54 (73%) presented B symptoms. Hodgkin lymphoma was diagnosed by histopathology and immunophenotyping. The disease stage was determined clinically according to the Ann Arbor system. The presence of B symptoms was defined as fever > 38°C, night sweats, and weight loss > 10% over a period of ≤ six months, as determined by reviewing patient charts and based on the classifications established by the referring physician.

On the basis of the histopathological analysis of the lymphoma, some of the patients were categorized as having classic Hodgkin lymphoma. The remaining patients were stratified by pathologic lymphoma subtype: nodular sclerosis; mixed cellularity; lymphocyte-predominant; or lymphocyte-depleted. Lymphomas were staged according to the Ann Arbor classification, and patients were characterized by age and gender.

### Image acquisition

Each patient underwent a three-dimensional PET/CT scan from skull base to mid-thigh approximately 60 min after injection of 370 MBq (10 mCi) of ^18^F-FDG. Images were obtained on a PET/CT scanner with time-of-flight technology (Discovery PET/CT 690; GE Healthcare, Milwaukee, WI, USA). The PET images were acquired for 3 min per bed position (15-cm slice thickness with a 3-cm overlap). The iterative technique with 24 subsets was used for PET image reconstruction in all studies. For attenuation correction and diagnostic purposes, we obtained non-contrast-enhanced CT transmission scans using the following parameters: current, 125 mAs; voltage, 120 kVp; gantry rotation, 0.5 s; pitch, 1.375; and axial slice thickness, 3.75 mm.

### Image analysis

**Figure 1:**
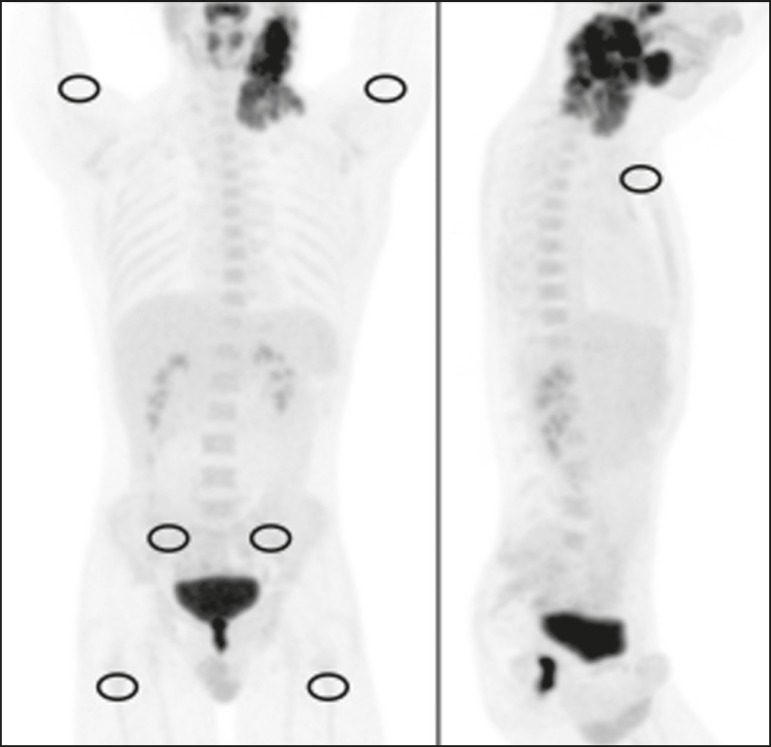
^18^F-FDG PET/CT study of a patient with Hodgkin lymphoma with ROIs drawn on the sternum, proximal thirds of the humeri, proximal thirds of the femora, and both iliac wings

As illustrated in Figure 1, elliptical regions of interest (ROIs), each measuring 2.5-3.0 cm at its greatest diameter, were drawn on the sternum, the proximal thirds of the humeri, the proximal thirds of the femora, and both iliac wings (totaling seven ROIs per patient). For all patients, the ROIs were drawn by the same nuclear physician, who was blinded to the symptom group.

### Statistical analysis

For each of the seven ROIs, the groups with and without B symptoms were compared, in terms of the maximum standardized uptake value (SUV_max_), with the Mann-Whitney U test. We also compared the two groups, in terms of the pathologic subtype, disease stage, patient age, and patient gender, using the Mann-Whitney U test (for patient age) or the chi-square test (for the remaining variables).

## RESULTS

The characteristics of the two groups of patients are presented in Table 1. As can be seen in the table, there was no statistically significant difference between the two groups in terms of age. We observed a predominance of the nodular sclerosis subtype in the B symptoms group. In addition, there was a tendency toward more advanced stages of lymphoma in the B symptoms group, with borderline significance (*p* = 0.12).

**Table 1:** Demographic and clinical characteristics of Hodgkin lymphoma patients with and without B symptoms.

		Patients	Patients	
		with	without	
	Total	B symptoms	B symptoms	
Characteristic	(n = 74)	(n = 54)	(n = 20)	*P*
Gender, n (%)				
Female	43 (58)	32 (59)	11 (55)	0.47
Male	31 (42)	22 (41)	9 (45)	
Age (years)				
Mean ± SD	35 ± 16	36 ± 16	32 ± 13	0.38
Range	16–80	16–80	16–66	
Subtype, n (%)				
Nodular sclerosis	37 (50)	32 (59)	5 (25)	
Unclassified	25 (34)	18 (33)	7 (35)	
Mixed cellularity	6 (8)	2 (4)	4 (20)	0.005
Lymphocyte-predominant	6 (8)	2 (4)	4 (20)	
Lymphocyte-depleted	0 (0)	0 (0)	0 (0)	
Lymphoma stage, n (%)				
I	2 (3)	0 (0)	2 (10)	
II	31 (42)	24 (44)	7 (35)	0.12
III	24 (32)	17 (32)	7 (35)	
IV	17 (23)	13 (24)	4 (20)	

SD, standard deviation.

**Figure 2:**
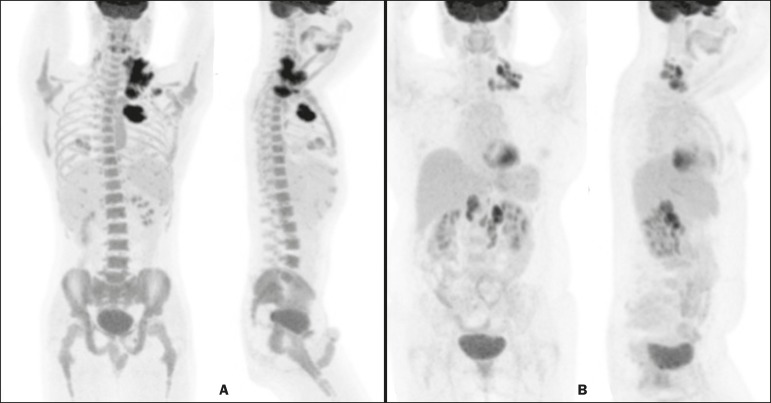
^18^F-FDG PET/CT studies of two patients-one with B symptoms (**A**) and one without (**B**)-showing that the bone marrow uptake of ^18^F-FDG was greater in the patient with B symptoms.

The mean, standard deviation, and range of the SUV_max_ for each of the seven ROIs are presented, by group, in Table 2, as are the corresponding *p*-values. For six of the seven ROIs, there were statistically significant differences between the two groups in terms of the SUV_max_, which was higher in the B symptoms group. There was also a tendency toward significantly higher SUV_max_ for the right iliac wing ROIs in the B symptoms group (*p* = 0.06). Examples of ^18^F-FDG PET/CT studies of patients with and without B symptoms are shown in Figure 2.

**Table 2:** SUV_max_ for each of the ROIs evaluated in Hodgkin lymphoma patients with and without B symptoms.

		Patients with	Patients without	
	Total	B symptoms	B symptoms	
ROI	(n = 74)	(n = 54)	(n = 20)	*P*
Sternum				
Mean ± SD	2.8 ± 1.3	3.0 ± 1.5	2.2 ± 0.6	0.01
Range	1.0–8.0	1.3–8.0	1.0–3.3	
Right humerus				
Mean ± SD	2.3 ± 1.3	2.6 ± 1.4	1.7 ± 0.8	0.01
Range	0.4–6.8	0.4–6.8	0.5–3.7	
Left humerus				
Mean ± SD	2.3 ± 1.4	2.5 ± 1.5	1.7 ± 0.8	0.03
Range	0.6–7.6	0.6–7.6	0.6–3.6	
Right femur				
Mean ± SD	2.5 ± 1.2	2.7 ± 1.2	2.0 ± 0.7	0.04
Range	0.7–7.0	0.7–7.0	0.8–3.5	
Left femur				
Mean ± SD	2.5 ± 1.1	2.7 ± 1.2	2.0 ± 0.7	0.03
Range	0.6–6.3	0.6–6.3	1.0–3.8	
Right iliac wing				
Mean ± SD	3.0 ± 1.2	3.2 ± 1.4	2.6 ± 0.7	0.06
Range	1.3–8.6	1.3–8.6	1.6–4.1	
Left iliac wing				
Mean ± SD	3.1 ± 1.2	3.3 ± 1.3	2.6 ± 0.7	0.02
Range	1.0–7.6	1.0–7.6	1.6–4.0	

SD, standard deviation.

## DISCUSSION

Recent studies conducted in Brazil have highlighted the importance of functional imaging with single-photon emission CT and PET/CT using ^18^F-FDG to improving the diagnosis of several diseases^(21-26)^. In the present cross-sectional study, we have demonstrated an association between the presence of B symptoms and greater benign bone marrow uptake of ^18^F-FDG in patients with Hodgkin lymphoma. Although the reasons for this association are unknown, it could be related to the production of cytokines in the tumor microenvironment. Neoplastic Hodgkin and Reed-Sternberg cells interact with reactive cells of the tumor microenvironment, and that interaction has been reported to be associated with high levels of cytokines^(27)^. In addition, the local production of those cytokines results in elevated systemic levels in the peripheral blood, which leads to the development of systemic symptoms and biochemical abnormalities that are correlated with disease prognosis^(28)^. It has also reported that interleukin (IL)-6 is the cytokine most closely associated with lymphopenia and B symptoms in lymphoma patients^(29)^, as well as that serum IL-6 levels are higher in Hodgkin lymphoma patients with B symptoms^(30)^. The levels of hepcidin, the expression of which is induced by IL-6, have been shown to be higher in patients with more aggressive disease characteristics, such as stage IV disease, B symptoms, and an International Prognostic Score > 2^(31)^. The levels of IL-9 have also been shown to correlate with B symptoms^(32)^. However, there have also been studies showing that stimulation of hematopoietic cytokines can cause a diffuse increase in bone marrow accumulation of ^18^F-FDG, mimicking bone marrow metastasis, on PET imaging^(33,34)^. In one study, conducted by Salaun et al.^(35)^, the degree of diffuse bone marrow uptake at the initial staging of Hodgkin lymphoma was correlated with the level of C-reactive protein, an inflammatory marker. The authors concluded that, although diffuse bone marrow uptake at the initial staging of Hodgkin lymphoma could be due to bone marrow involvement, it was more likely due to inflammatory changes in the bone marrow. Therefore, in the neoplastic cell microenvironment, the increased diffuse bone marrow uptake of ^18^F-FDG in Hodgkin's lymphoma patients with B symptoms could be mediated by the increased production of cytokines, which are inflammatory modulators. It is noteworthy that those authors also found a statistically significant association between the SUV_max_ in the sacrum and the presence of B symptoms. Therefore, their findings support the results of our study, in which we analyzed a large number of bone regions.

Our study has certain limitations. The statistically significant differences between our groups of patients with and without B symptoms, in terms of the lymphoma subtypes, have the potential to confound the results. The predominance of the nodular sclerosis subtype in the B symptoms group is not an unexpected finding, a previous study having shown a difference between patients with and without B symptoms in terms of the prevalence of Hodgkin lymphoma subtypes^(36)^. However, that difference does not alter our conclusions, because our hypothesis was that there would be an association, rather than a causal relationship, between B symptoms and increased ^18^F-FDG uptake in bone marrow. Another potential limitation of our study is related to the accuracy of the information regarding B symptoms. Information about B symptoms was obtained from patient charts, based on the reporting of the referring physicians, and might therefore be inaccurate. However, there is no reason for such inaccuracies to occur in one particular direction (favoring the presence or absence of symptoms) and they tend to diminish the strength of an association rather than increasing it. The fact this was a cross-sectional study could also be seen as a limitation, because cross-sectional analyses use data collected for other purposes and are often unable to include all data on confounding variables that potentially affect the relationship between cause and effect. Nevertheless, as previously mentioned, we did not hypothesize a causal relationship between B symptoms and benign ^18^F-FDG bone marrow uptake. Therefore, because we believe that B symptoms and benign bone marrow uptake of ^18^F-FDG could both be attributed to upregulation of cytokine production, a cross-sectional analysis seems well suited to testing our hypothesis.

## CONCLUSION

In our sample of patients with Hodgkin lymphoma, the presence of B symptoms was associated with a benign diffuse increase in the uptake of ^18^F-FDG in bone marrow.
